# Choroidal thickness as a possible predictor of non-response to intravitreal bevacizumab for macular edema after retinal vein occlusion

**DOI:** 10.1038/s41598-023-27753-7

**Published:** 2023-01-09

**Authors:** Wissam Aljundi, Florian Gradinger, Achim Langenbucher, Haris Sideroudi, Berthold Seitz, Alaa Din Abdin

**Affiliations:** 1 Department of Ophthalmology, Saarland University Medical Center UKS, Kirrberger Street 100, Building 22, 66421 Homburg, Saar Germany; 2 Institute of Experimental Ophthalmology, Saarland University, Homburg, Saar Germany

**Keywords:** Clinical trials, Retinal diseases

## Abstract

To evaluate outcomes of intravitreal bevacizumab (IVB) treating macular edema (ME) after retinal vein occlusion (RVO) following pro re nata (PRN) regimen and investigate potential predictors of non-response. Retrospective analysis of 126 treatment-naive eyes with ME after RVO. Eyes were treated initially with IVB of 1.25 mg/ml. Therapy was switched in case of non-response. Outcome measures included best-corrected visual acuity (BCVA) and central macular thickness (CMT), which were recorded over 4 years of treatment. BCVA improved significantly during first 2 years. CMT decreased significantly during the 4-year follow-up period. Switching was required in 42 eyes (33%). 34 eyes (26.9%) were switched to steroids, while 8 eyes (6.3%) were switched to other anti-VEGF due to diagnosed glaucoma. Switching occurred after 12.4 ± 8.3 months and an average of 8 ± 4.1 IVBs. Compared with the treatment-responsive group, the treatment-unresponsive group had significantly worse BCVA, higher CMT and subfoveal choroidal thickness (SFCT) at baseline. Treatment IVB following PRN regimen showed significant functional and anatomic improvement in patients with ME after RVO. Switching was required in more than one third of eyes. Higher baseline SFCT could be considered as predictor for non-response to such therapy and thus an indicator of early switching.

## Introduction

Retinal vein occlusion (RVO) is the second most common vascular cause of blindness after diabetic retinal disease, with varying incidence (0.7–1.6%)^1–3^.

Depending on the location of the occlusion, RVO can be classified in 2 forms: central retinal vein occlusion (CRVO) and branch retinal vein occlusion (BRVO). CRVO was first described by von Graefe in 1859 and represents a sight-threatening ophthalmologic emergency with marked visual loss and usually a poor prognosis^4,5^.

BRVO is the most common retinal vein occlusion and was first described by Leber in 1877^6,7^. BRVO can be subclassified into major BRVO and macular BRVO. The hemi-retinal vein occlusion (HRVO) is usually considered as a subtype of CRVO^8^.

Cystoid macular edema (CME) is the most common vision-threatening complication of RVO and its pathogenesis is not fully understood^9^. The increased transudation of blood products into the retina due to increased intraluminal pressure after occlusion in the acute phase leads to an increase in interstitial retinal pressure, which prompts edema formation and leads to ischemia and neovascularization (NV). In addition, inflammation plays a significant role in RVO. Many inflammatory molecules (such as vascular endothelial growth factor (VEGF) and interleukin 6 and 8) are found to be increased in vitreous cavity after RVO^9–11^.

RVO is a multifactorial disease that has systemic as well as ocular risk factors. Age represents the most important systemic risk factor (older than 65 years in more than 50% of cases). In addition, microangiopathy due to arterial hypertension, diabetes mellitus or hyperlipidemia is also an influential risk factor^12^. Glaucoma, decreased ocular perfusion are considered as ocular risk factors.

PDS is characterized by an alteration in choroidal structure manifested by an increase in choroidal thickness as well as an attenuation of the choriocapillaris and Sattler’s layer overlying focally or generally dilated choroidal veins in Haller’s layer (pachyvessels)^13,14^.

There is no causal treatment of RVO. For this reason, treatment is limited to the management of the complications of RVO such as retinal ischemia, neovascular glaucoma (NVG), retinal detachment and CME^15^.

For the treatment and prevention of NV and NVG, panretinal photocoagulation (PRP) is widely recommended^5,16^.

Many published papers investigated the use of intravitreal injection therapy (IVI) with both anti-VEGF agents and steroids and concluded that this use has a major benefit on macular anatomy and function in CRVO and BRVO in both short and long term. Based on this papers, ranibizumab, aflibercept, dexamethasone implant have been approved for the treatment of ME following RVO in Europe and the US^17–20^.

Since the altered structure of the choroid has recently been recognized as a risk factor for the development of RVO^13^, and because many previous published papers found an increased choroidal thickness in RVO eyes compared with contralateral healthy eyes^21–24^, the main aim of our study was to investigate whether an increased subfoveal choroidal thickness (SFCT), as well as other factors at baseline, may affect the response to initial IVI therapy with bevacizumab in patients with RVO. Moreover, we evaluated the long-term functional and anatomic outcomes of this treatment.

## Materials and methods

**Figure 1:**
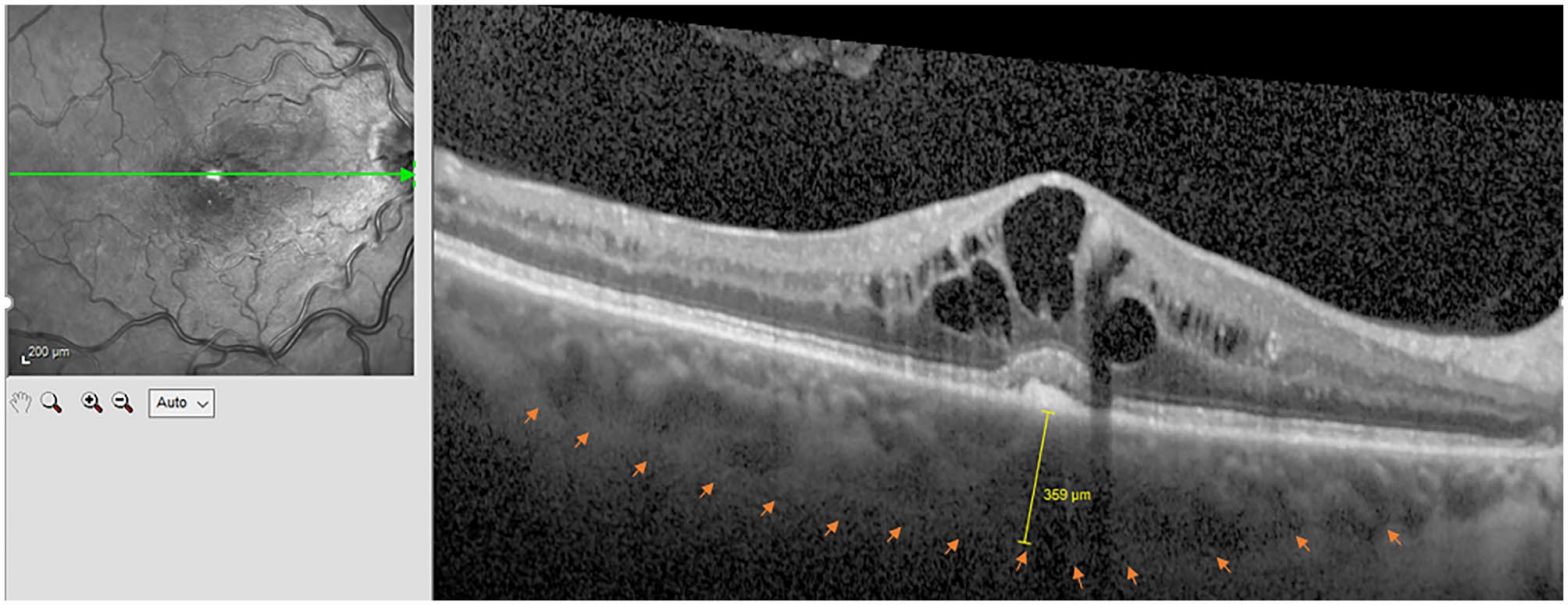
Measurement of subfoveal choroidal thickness (SFCT) using enhanced depth imaging optical coherence tomography (EDI-OCT) in a male patient of our study with central retinal vein occlusion. The inner surface of the sclera is indicated with the arrows. SFCT was 359 µm.

We retrospectively evaluated patient data from our department's electronic RVO database. For each patient, the best-corrected visual acuity (BCVA) was assessed in decimal and converted to logMAR. In addition, fluorescein angiography (FA), spectral domain optical coherence tomography (SD-OCT), and enhanced depth imaging OCT (EDI-OCT) were performed in all patients using Spectralis HRA/OCT (Heidelberg Engineering, Heidelberg, Germany). EDI-OCT images were taken by different technicians and analyzed in a masked manner by one experienced ophthalmologist. SFCT (in µm) was defined as the vertical distance from the hyperreflective line of Bruch's membrane to the hyperreflective line of the inner surface of the sclera. SFCT was then measured using EDI-OCT as explained, which has high intra- and interobserver reproducibility (Fig. 1)^25^.

In this study, we included 126 naive eyes (out of 126 patients) treated initially with intravitreal bevacizumab from January 2016 to June 2020 due to CME secondary to BRVO (80 eyes) or CRVO (46 eyes).

Indication for injection:

- ME secondary to CRVO or BRVO at least 3 months after the occlusion event.
- BCVA (logMAR) < 1.4 and > 0.

Reinjection was performed in case of persistence or increase of ME identified by OCT (CMT > 250 µm) or decreased BCVA by one or more lines on Snellen chart compared to the last visit.

Eyes were treated with three initial intravitreal bevacizumab injections of 1.25 mg/ml at monthly intervals (upload phase). Retreatment was then applied depending on reactivity according to a Pro re nata (PRN) regimen. All injections were performed in a designated intravitreal injection center in our Department of Ophthalmology at the Saarland University Medical Center^26^. Due to the lack of a clear definition of response to intravitreal therapy for ME in eyes with RVO in previously published papers, we defined response as a decrease in central macular thickness (CMT) of ≥ 10% or an improvement in BCVA of one or more lines in Snellen chart.

The studied eyes were divided into 2 main groups according to response to initial therapy with bevacizumab: group 1 for eyes that showed a response during its observation period and did not require switching to another agent, and group 2 for eyes that were switched to another agent because of non-response to initial therapy.

In group 2, we distinguished between 2 types of non-response to bevacizumab: primary non-response, if observed at the end of upload phase (subgroup a), and late non-response, if observed at any other time within the treatment period after an initial response (subgroup b).

In case of non-response to bevacizumab, therapy was switched to steroids: except in patients with history of glaucoma as an absolute contraindication. In this case the therapy was switched to another anti-VEGF agent. In addition, patients with elevated intraocular pressure after first switching “steroid responders” were switched to other anti-VEGF agents during follow-up.

Outcome measures included changes in BCVA, CMT. All outcome measures were recorded before treatment, 6 months, 1 year, 2 years, 3 years and 4 years after treatment and were analyzed and compared separately between eyes with BRVO and CRVO, as well as between the two main and subgroups to define potential predictors of non-response to intravitreal bevacizumab.

### Statistical analysis

Data were collected using Microsoft Excel 2010 (Microsoft Corporation, Redmond, WA, USA) and analyzed using SPSS version 27 (SPSS Inc, Chicago, IL, USA). We used the Mann–Whitney U test for non-normally distributed variables and the t-test for normally distributed variables. In order to compare frequencies, we used the chi-square (χ^2^) test. Multivariate regression analysis was performed in order to assess potential predictors of non-response to intravitreal therapy. This analysis could not be performed for eyes with BRVO and CRVO separately, as the statistical power would then no longer be maintained. The mean values ± SD of the data were presented, and differences were considered significant if P < 0.05.

### Institutional review board statement

This study was approved by the Ethics Committee of the Medical Association of Saarland, Germany (Nr. 123/20). All procedures performed in this study were in accordance with the ethical standards of the institutional research committee and with the 1964 Helsinki declaration and its later amendments or comparable ethical standards. This article does not contain any studies with animals performed by any of the authors.

### Informed consent statement

Since bevacizumab has not yet been approved for the treatment of CME following RVO, all patients were informed in detail about this off-label therapy and signed a written consent form. For this type of retrospective study, formal consent is not required.

## Results

A total of 126 eyes were included in this study (80 eyes with BRVO and 46 eyes with CRVO). Based on the Central Retinal Vein Occlusion Study (CVOS) an ischemic RVO (with an amount of capillary non-perfusion of at least 10 disc areas^16^) was found in a total of 21 cases (9 BRVO and 12 CRVO).

The minimum follow-up time for all 126 eyes was 1 year after the first IVI. Of these, 63 eyes were followed-up for 2 years, 50 eyes for 3 years and 33 eyes for 4 years after the first IVI. The number of injections per eye was 6.8 ± 2.4 in the first year, 4.6 ± 2.6 in the second year, 4.3 ± 2.8 in the third year, and 4.1 ± 2.8 in the fourth year of treatment.

While 84 eyes continued to receive intravitreal bevacizumab for the entire follow-up duration (*group 1*), therapy with intravitreal bevacizumab had to be switched to another agent in 42 eyes due to non-response (*group 2*). The baseline characteristics and number of IVIs in total eyes, as well as in both groups are listed in Tables 1 and 2.

**Table 1:** The baseline characteristics of study eyes and both groups.

Baseline characteristics (means ± SD)				
Variable	n = 126	Response (Group 1, N = 84)	Non-response (Group 2, N = 42)	p-value
Male/female	43%/57%	50%/50%	27%/73%	> 0.05
Right/left	57%/43%	58%/42%	59%/41%	> 0.05
Age	73 ± 12	70 ± 13	74 ± 10	> 0.05
DM	19%	16%	20%	> 0.05
Heart diseases	26%	28.9%	22%	> 0.05
Lung diseases	6%	5%	8%	> 0.05
Kidney diseases	9%	5%	16%	> 0.05
Arterial hypertension	59%	51%	70%	> 0.05
Thyroid diseases	16%	15%	17%	> 0.05
Smoking	3%	2%	4%	> 0.05
CRVO/BRVO	38%/62%	36%/64%	33%/67%	> 0.05
Phakic/pseudophakic	56%/44%	54%/46%	58%/42%	> 0.05
BCVA (logMAR)	0.54 ± 0.3	0.53 ± 0.29	0.57 ± 0.49	**0.025**
CMT (µm)	539 ± 191	520 ± 189	591 ± 191	**0.001**
SFCT (µm)	281 ± 70	271 ± 36	301 ± 70	**0.03**
SFCT > 300 µm	38%	28%	55%	**0.003**

Significant values are in bold.

Group 1 (response group, n = 84): eyes that showed a therapeutic response to intravitreal bevacizumab.

Group 2 (non-response group, n = 42): eyes that did not show a therapeutic response to intravitreal bevacizumab.

*SD* standard deviation, *DM* diabetes mellitus, *BCVA* best-corrected visual acuity, *CMT* central macular thickness, *SFCT* subfoveal choroidal thickness, *CRVO* central retinal vein occlusion, *BRVO* branch retinal vein occlusion.

**Table 2:** The baseline characteristics of both subgroups.

Variable	Early non-response (subgroup a, N = 14)	Late non-response (subgroup b, N = 28)	p-value
Male/female	35%/65%	30%/70%	> 0.05
Right/left	55%/45%	60%/40%	> 0.05
Age	71 ± 10	73 ± 11	**0.02**
DM	7%	14%	> 0.05
Heart diseases	21.4%	25%	> 0.05
Lung diseases	7%	3%	> 0.05
Kidney diseases	7%	3%	> 0.05
Arterial hypertension	85%	78%	> 0.05
Thyroid diseases	14%	28%	> 0.05
Smoking	7%	3%	> 0.05
CRVO/BRVO	21%/79%	39%/61%	> 0.05
Phakic/pseudophakic	50%/50%	55%/46%	> 0.05
BCVA (logMAR)	0.41 ± 0.21	0.32 ± 0.20	> 0.05
CMT (µm)	451 ± 86	473 ± 132	> 0.05
SFCT (µm)	312 ± 51	284 ± 95	> 0.05
SFCT > 300 µm	79%	46%	**0.04**

Significant values are in bold.

Subgroup a (early non-response group, n = 14): eyes that showed non-response to intravitreal bevacizumab at the end of upload phase.

Group 2 (late non-response group, n = 28): eyes that showed late non-response to intravitreal bevacizumab during follow-up after an initial response.

*SD* standard deviation, *DM* diabetes mellitus, *BCVA* best-corrected visual acuity, *CMT* central macular thickness, *SFCT* subfoveal choroidal thickness, *CRVO* central retinal vein occlusion, *BRVO* branch retinal vein occlusion.

While 14 eyes showed an early non-response at the end of the upload phase (*subgroup a*), late non-response occurred in 28 eyes (*subgroup b*). An analysis of the baseline characteristics and possible non-response predictors in both subgroups is shown in Table 3. Switching occurred after 3 months in subgroup a (after 3 IVIs) and after 16 ± 6 months in subgroup b (after 12 ± 3 IVIs).

**Table 3:** The number of injections in both study eyes.

Number of IVIs				
	n = 126	Group 1 (N = 84)	Group 2 (N = 42)	p-value
1. y	6.8 ± 2.4	6.74 ± 2.39	7.1 ± 2.45	> 0.05
2. y	4.6 ± 2.6	4.96 ± 2.61	4.23 ± 2.59	> 0.05
3. y	4.3 ± 2.8	4.68 ± 2.73	3.82 ± 2.6	> 0.05
4. y	4.1 ± 2.8	3.62 ± 2.4	4.63 ± 3.1	> 0.05

Group 1 (response group, n = 84): eyes that showed a therapeutic response to intravitreal bevacizumab.

Group 2 (non-response group, n = 42): eyes that did not show a therapeutic response to intravitreal bevacizumab.

**Figure 2:**
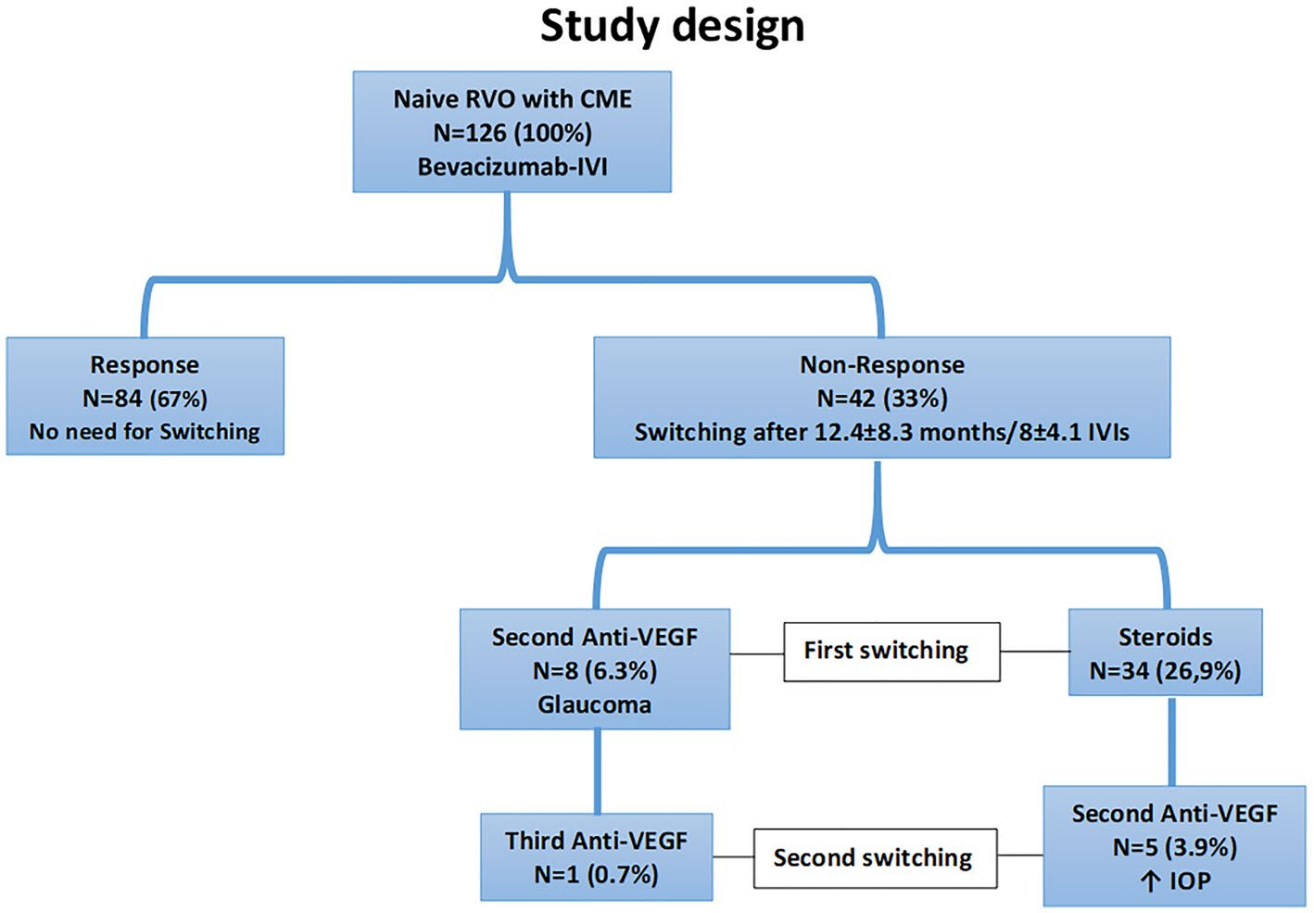
An overview of our study design and the therapy course: a total of 126 eyes were included in the study and received intravitreal Bevacizumab: 84 eyes continued to receive intravitreal bevacizumab for the entire follow-up duration (*group 1*), therapy with intravitreal bevacizumab had to be switched to another agent in 42 eyes due to non-response (*group 2*). While 14 eyes showed an early non-response at the end of the upload phase (*subgroup a*), late non-response occurred in 28 eyes (*subgroup b*). This first switching was to steroids in 34 eyes and to a second anti-VEGF agent in 8 eyes because of a pre-diagnosed glaucoma. A second switching occurred because of a renewed non-response from the second to a third anti-VEGF agent in one eye and from steroids to a second anti-VEGF agent in 5 eyes because of steroid response. *RVO* retinal vein occlusion, *CME* cystoid macular edema, *IVI* intravitreal injection, *anti-VEGF* anti vascular endothelial growth factor, *IOP* intraocular pressure.

An overview of our study design and the therapy course is shown in Fig. 2.

This first switching was either to steroids in 34 eyes or to a second anti-VEGF agent in 8 eyes because of a pre-diagnosed glaucoma. A second switching occurred because of a renewed non-response: either from a second to a third anti-VEGF agent in one eye or from steroids to a second anti-VEGF agent in 5 eyes because of steroid response with elevated intraocular pressure.

Baseline SFCT of contralateral healthy eyes (in µm) was significantly lower compared to affected eyes: 218 ± 68 vs. 244 ± 66 in eyes with BRVO (p = 0.01), 207 ± 55 vs. 249 ± 76 in eyes with CRVO (p = 0.04) and 215 ± 64 vs. 212 ± 66 for the entire cohort (p = 0.02).

We could not find a statistically significant correlation between the decrease in SFCT and the improvement of BCVA at any follow-up (range, r = − 0.210–1, p > 0.05).

In addition, prevalence of increased SFCT > 300 µm was significantly higher in group 2 (p = 0.003) compared to group 1 (response group), as well as in subgroup a (early non-response group) (p = 0.04) compared to subgroup 2 (late non-response group).

The Functional and morphological changes are shown in Table 4.

**Table 4:** Functional and morphological changes.

Functional and morphological changes (means ± SD)				
Type		BCVA (logMAR)	CMT (µm)	SFCT (µm)
BRVO (n = 80)	Baseline	0.46 ± 0.28	479 ± 169	244 ± 66
	6 months	0.32 ± 0.25*	344 ± 90*	213 ± 66*
	1 year	0.35 ± 0.32*	330 ± 96*	233 ± 61
	2 years	0.41 ± 0.31*	356 ± 135*	241 ± 72
	3 years	0.46 ± 0.28	323 ± 93*	256 ± 79
	4 years	0.41 ± 0.29*	405 ± 136*	241 ± 79
CRVO (n = 46)	Baseline	0.66 ± 0.29	614 ± 197	249 ± 76
	6 months	0.52 ± 0.32*	405 ± 173*	210 ± 66*
	1 year	0.50 ± 0.36*	511 ± 284*	233 ± 64
	2 years	0.56 ± 0.31*	395 ± 212*	270 ± 85
	3 years	0.46 ± 0.32*	333 ± 107*	252 ± 107
	4 years	0.33 ± 0.21*	448 ± 140*	247 ± 56
All eyes with RVO (n = 126)	Baseline	0.54 ± 0.3	539 ± 191	246 ± 69
	6 months	0.39 ± 0.29*	378 ± 140*	212 ± 66*
	1 year	0.38 ± 0.2*	373 ± 151*	233 ± 61
	2 years	0.44 ± 0.3*	374 ± 175*	251 ± 77
	3 years	0.5 ± 0.3	336 ± 105*	255 ± 88
	4 years	0.47 ± 0.3	377 ± 126*	243 ± 71

Significant values are in bold.

*SD* standard deviation, *BCVA* best-corrected visual acuity, *CMT* central macular thickness, *SFCT* subfoveal choroidal thickness, *RVO* retinal vein occlusion, *BRVO* branch retinal vein occlusion, *CRVO* central retinal vein occlusion.

*p-value < 0.05 when compared to baseline.

Eyes with ischemic CRVO showed worse BCVA (logMAR) compared with eyes with non-ischemic CRVO (Table 5). A statistically significant difference was found at baseline, 6 months, 1 year, and 4 years follow-ups. Otherwise, the difference was not statistically significant at 2- and 3-years follow-ups.

**Table 5:** Changes of BCVA in eyes with ischemic and non-ischemic CRVO.

Changes of BCVA in logMAR (means ± SD)			
	Non-ischemic CRVO	Ischemic CRVO	p-value
Baseline	0.59 ± 0.27	0.81 ± 0.29	**< 0.01**
6 months	0.46 ± 0.31	0.74 ± 0.27	**< 0.01**
1 year	0.41 ± 0.32	0.79 ± 0.33	**< 0.01**
2 years	0.49 ± 0.29	0.78 ± 0.29	> 0.05
3 years	0.41 ± 0.31	0.74 ± 0.24	> 0.05
4 years	0.39 ± 0.15	0.84 ± 0.21	**< 0.01**

Significant values are in bold.

*SD* standard deviation, *BCVA* best-corrected visual acuity, *CRVO* central retinal vein occlusion.

**Figure 3:**
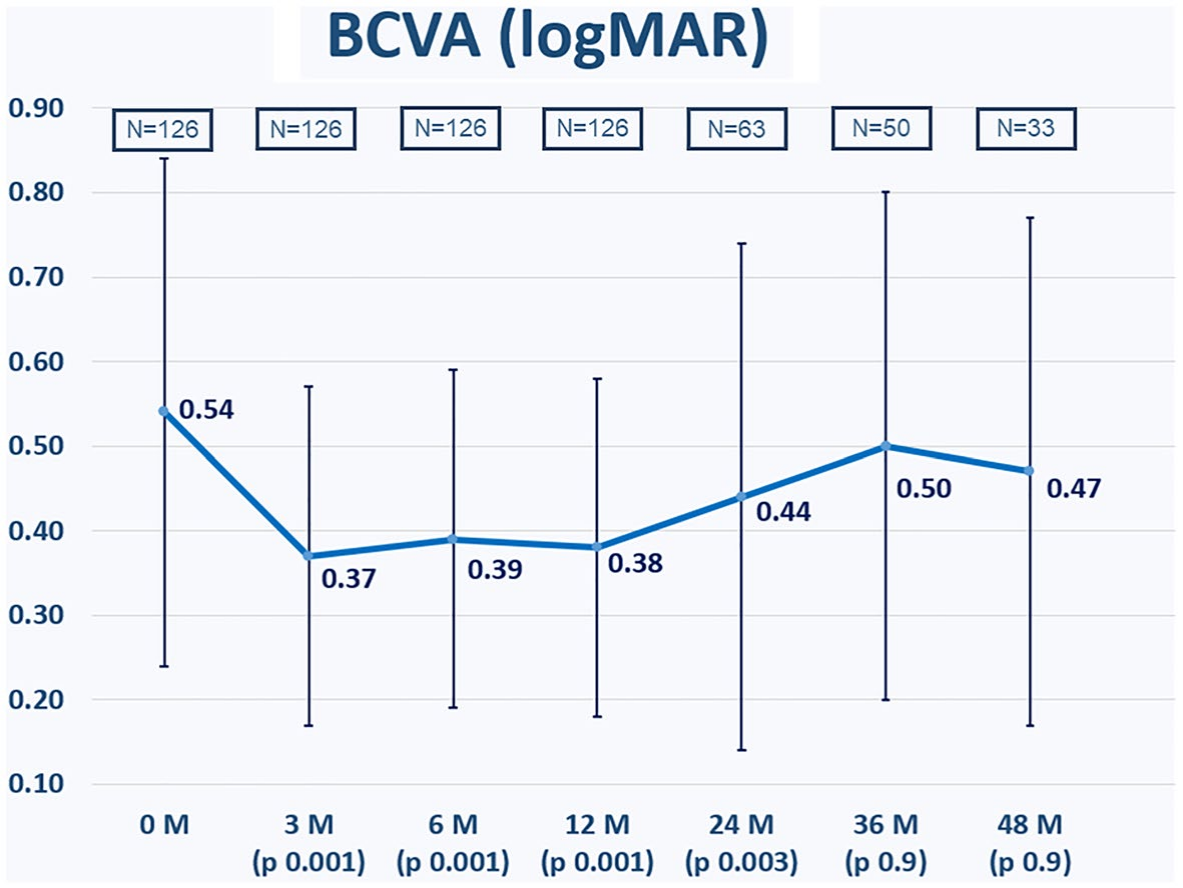
The change in best-corrected visual acuity (BCVA, logMAR) during the study observation period. p values refer to statistical differences between each time points and baseline: BCVA improved significantly at 6 months (p = 0.001), 1 year (p = 0.001), and 2 years (p = 0.003) but not significantly after 3 years (p = 0.9) and 4 years (p = 0.9) after the first intravitreal injection.

The change in BCVA is shown in Fig. 3. BCVA improved significantly at 6 months (p = 0.001), 1 year (p = 0.001), and 2 years (p = 0.003) but not significantly after 3 years (p = 0.9) and 4 years (p = 0.9) after the first IVI.

**Figure 4:**
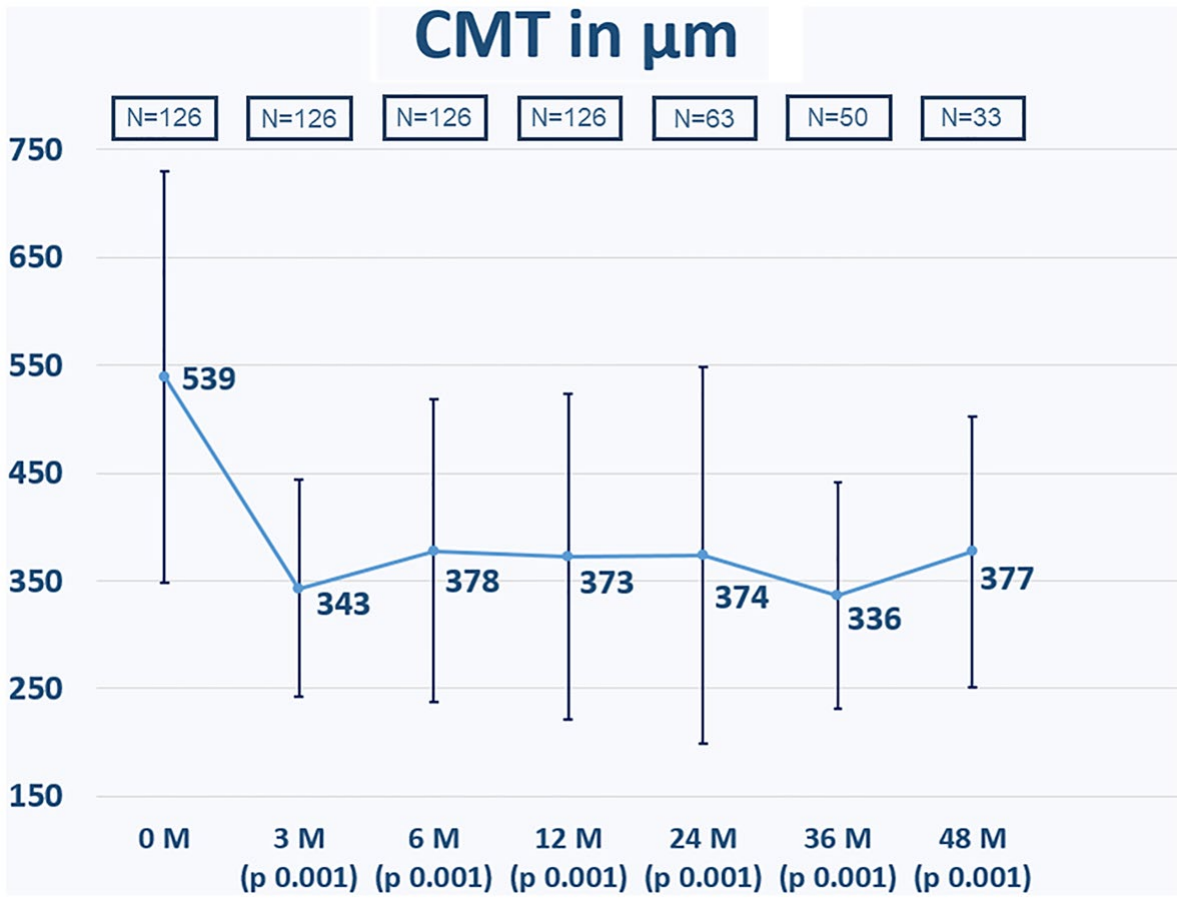
The change in central macular thickness (CMT, µm) during the study observation period. p values refer to statistical differences between each time points and baseline: CMT decreased significantly at all time points (p = 0.001).

The change in CMT is shown in Fig. 4. CMT decreased significantly in all time points compared to baseline (p = 0.001).

**Figure 5:**
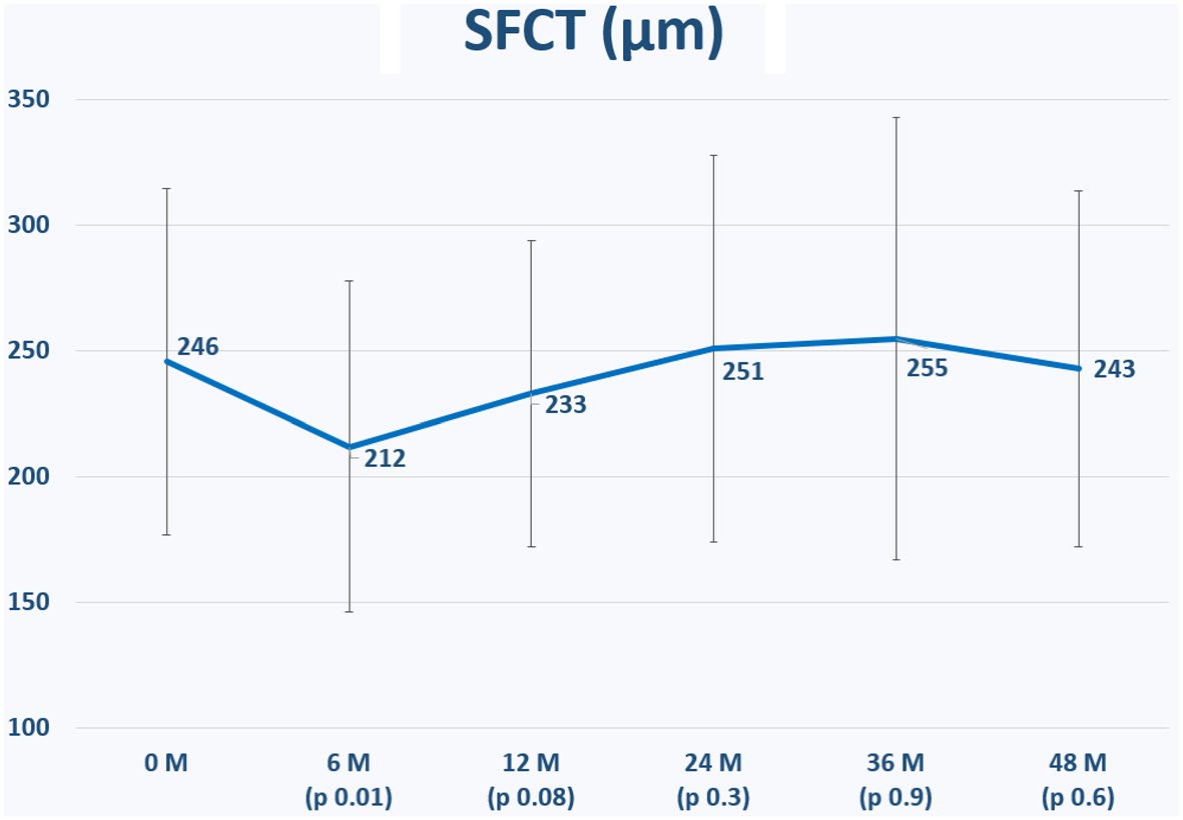
The change in subfoveal choroidal thickness (SFCT, µm) during the study observation period. p values refer to statistical differences between each time points and baseline: SFCT improved significantly only 6 months after the first intravitreal injection (p = 0.01).

The change in SFCT is shown in Fig. 5. SFCT decreased significantly at 6 months (p = 0.01), but not significantly at 1 year (p = 0.08), 2 years (p = 0.3), 3 years (p = 0.9) and 4 years (p = 0.6) after the first IVI.

At baseline, eyes in group 2 (non-response group) showed significantly worse BCVA (p = 0.025), significantly higher CMT (p = 0.001) and significantly higher SFCT (p = 0.03) compared to group 1 (response group)^14^.

The mean age was significantly lower in subgroup a (early non-response group) compared to subgroup b (late non-response group) (p = 0.02).

No ocular or systemic adverse events were observed.

## Discussion

In this study, we present our long-term results for the treatment of CME following RVO with bevacizumab. The beneficial effect of anti-VEGF agents for the treatment of CME after RVO is supported by many papers in the literature^27,28^. However, the major multicenter trials focus on aflibercept and ranibizumab^17,29–31^. Worldwide, bevacizumab is not approved for the treatment of macular edema in many diseases such as age-related macular degeneration (AMD), diabetic macular edema and RVO and is referred to as "off-label drug". The reason for this is that this use is not "evidence-based" and not supported by large clinical trials. Nevertheless, bevacizumab is one of the most commonly used anti-VEGF agents in real-life settings, as it is significantly less expensive and similarly effective compared to the other approved drugs mentioned above^32^. The problem with the intravitreal use of bevacizumab arose from the infrastructural and hygienic difficulty of properly distributing this originally large-volume anticancer drug in small volumes for intravitreal use, especially after many published papers had reported cases of endophthalmitis outbreak as a result of this use^33–36^. In our department bevacizumab is prepared under strict hygienic measures by experienced staff at our internal pharmacy and then within a minimum period of time injected at our standardized center^26^. This did not lead, as mentioned in the results section, to any relevant cases of adverse events including endophthalmitis.

Since it is known that anti-VEGF therapy has a positive impact on the course and final outcome of CME after RVO^37,38^, it is, however, not a prerequisite that every patient will benefit from this treatment. This is the main point why determining possible baseline characteristics or predictors is important in order to make an early switching and thus avoid additional unnecessary injections.

Similar to already published papers^39–41^, we found that eyes with worse BCVA, as well as higher CMT at baseline were significantly more likely to show non-response to initial intravitreal bevacizumab.

In addition, we found that an increase in baseline SFCT (> 300 µm) may be a predictive factor of early, as well as late, non-response to initial intravitreal bevacizumab.

A "healthy" choroid is essential for the development as well as the function of the retina, since the choroid regulates the metabolism of the retinal pigment epithelium (RPE) and the outer retina. In addition, the choroid plays an important role in the growth of the eye and thus possibly in the development of myopia, where a thin choroid is present^42,43^. On the other hand, an isolated increase in SFCT (> 300 µm) usually is referred to as "pachychoroid". This should be distinguished from the pathological increase in choroidal thickness, or the so-called "pachychoroid disease spectrum (PDS)"^44^. This term was first discussed in 2013 and is defined as a pathological and persistent usually bilateral increase in choroidal thickness, often with dilated choroidal vessels and other structural choroidal changes^14,45^.

In this context, the role of the choroid in the development of retinal vascular impairment was investigated. In 2016, Nagia et al. found that an increase in peripapillary choroidal thickness plays a role in the pathogenesis of non-arteritic anterior ischemic optic neuropathy (NAION). This was correlated to a possible compartment syndrome, in which the axons and their blood supply are strained with fluctuations in choroidal volume within the narrow central cup^46^.

Alteration of the choroid as a result of anti-VEGF intravitreal therapy for RVO, as well as other diseases such as neovascular AMD has been widely investigated in the literature^23,47–51^. These studies delivered varying conclusions. For example, Tsuiki et al. and Coban et al. found a significant difference in SFCT between affected and fellow eyes, as well as before and after intravitreal anti-VEGF therapy in eyes with RVO^24,50^. This difference could not be detected by Park et al.^47^.

We found a significant decrease in SFCT only in the first 6 months after the first injection in eyes with BRVO and CRVO. Nevertheless, this significant decrease was no longer detectable thereafter. This result could neither be explained by those previously published papers nor compared with their results. The reason for this might be the different follow-up period (up to 4 years in the present study and less than one year in the previous studies).

An interesting study by Tag et al. even argued that the decrease in SFCT occurs immediately to shortly after anti-VEGF injection in RVO, before the choroidal thickness may restore. Larger multicenter studies with several patients and longer follow-up are necessary to provide a clear conclusion regarding choroidal alteration following anti-VEGF therapy in RVO, as well as in other diseases.

We could not find a statistically significant correlation between the decrease in SFCT and the improvement of BCVA at any follow-up (range, r = − 0.210–1, p > 0.05).

Unlike our study, Rayess et. al found in 2016, as well as in 2019, that a thicker baseline SFCT is a positive predictor of a functional response to intravitreal therapy of various anti-VEGF agents in terms of a visual gain of more than 2 Snellen lines in both CRVO and BRVO eyes^52,53^. This discrepancy in the results could be explained by the difference in study design between the studies mentioned and ours, especially regarding the definition of “response”. The anatomical-based response in our study could have resulted in some eyes ending up in the non-responder group, even though they could have had a functional response according to the definition of Rayess et al., but showed an insufficient decrease in CMT according to our definition.

Okamoto et al. investigated the influence of SFCT on the outcome of intravitreal therapy with ranibizumab in 32 eyes with BRVO and divided the patients into 2 groups: resolving group and recurrent group. Similar to our study design, the grouping was anatomic-based and consistent with our results, Okamoto et al. found that the choroid was significantly thicker in the recurrent group than in the resolving group, both at baseline and at all time points after intravitreal bevacizumab^54^.

We found that the eyes with RVO had significantly higher SFCT compared with the fellow contralateral eyes. Thus, this increase in SFCT could be explained by the presence of RVO and not as a manifestation of an undiagnosed disorder of PDS, where the increased SFCT typically appears bilateral^14^. This result is supported by important previously published papers^21–24^. Moreover, these papers described a significant decrease in SFCT after intravitreal therapy with ranibizumab or bevacizumab.

The role of the choroid in the development of RVO was investigated in a recent study on 312 eyes. This study suggests that patients with RVO have a higher SFCT and a higher incidence of PDS. Therefore, the pachychoroid might be a risk factor for RVO or share risk factors with RVO^13^.

Within the first 24 months, we observed a significant decrease in CMT associated with a significant improvement in BCVA. From this point on, a discrepancy between CMT and BCVA appeared, as CMT decreased significantly at each time point until the end of the 48-month follow-up without significant improvement of BCVA. This has been previously reported in the literature and could be explained by the fact that retinal ischemia prevents the improvement of BCVA despite the significant decrease of CMT^55,56^.

We did not observe any relevant adverse events regarding initial therapy with bevacizumab. However, within the 34 eyes switched to steroids, 5 eyes (14.7%) showed ocular hypertension (OHT, IOP > 25 mmHg) and were switched again to another anti-VEGF agent. OHT after both dexamethasone implant and intravitreal triamcinolone has been studied in the literature. Chin et al. reported that OHT was observed in 14 of the 59 eyes studied (26.9%)^57^. In 2020, Storey et al found that OHT occurred in 14 of the 106 eyes studied (13.2%)^58^.

The main potential limitations of our study were the retrospective nature of the work, a relatively small population from a single medical center, and the use of decimal visual acuity as opposed to ETDRS vision charts.

In conclusion we report, that intravitreal bevacizumab following PRN regimen showed significant functional and anatomic improvement in patients with CME after RVO. A therapy switching was required in more than one third of eyes. Higher SFCT at baseline could be considered as predictor for non-response to such therapy.

## Data Availability

The datasets generated during and/or analyzed during the current study are available from the corresponding author (Aljundi W.) on reasonable request.
